# Epstein - Barr virus latent membrane protein 1 suppresses reporter activity through modulation of promyelocytic leukemia protein-nuclear bodies

**DOI:** 10.1186/1743-422X-8-461

**Published:** 2011-10-05

**Authors:** Mark D Sides, Gregory J Block, Reid W Chadwick, Bin Shan, Erik K Flemington, Joseph A Lasky

**Affiliations:** 1 Department of Medicine, Section of Pulmonary Disease and Critical Care, Tulane University School of Medicine, 1430 Tulane Avenue, New Orleans, LA, 70112, USA; 2 Department of Pathology, Tulane University School of Medicine, 1430 Tulane Avenue, New Orleans, LA, 70112, USA; 3 University of Washington Institute for Stem Cell and Regenerative Medicine, 815 Mercer Street, Seattle, WA, 98108, USA

**Keywords:** Latent Membrane Protein 1, Epstein - Barr virus, reporter assay, promyelocytic leukemia protein, promyelocytic leukemia nuclear bodies, arsenic trioxide

## Abstract

The Epstein-Barr virus (EBV) encoded Latent Membrane Protein 1 (LMP1) has been shown to increase the expression of promyelocytic leukemia protein (PML) and the immunofluorescent intensity of promyelocytic leukemia nuclear bodies (PML NBs). PML NBs have been implicated in the modulation of transcription and the association of reporter plasmids with PML NBs has been implicated in repression of reporter activity. Additionally, repression of various reporters in the presence of LMP1 has been noted. This study demonstrates that LMP1 suppresses expression of reporter activity in a dose responsive manner and corresponds with the LMP1 induced increase in PML NB intensity. Disruption of PML NBs with arsenic trioxide or a PML siRNA restores reporter activity. These data offer an explanation for previously conflicting data on LMP1 signaling and calls attention to the possibility of false-positives and false-negatives when using reporter assays as a research tool in cells expressing LMP1.

## Background

The Epstein -Barr Virus (EBV) encoded Latent Membrane Protein 1 (LMP1) is required for the immortalization of B-cells by EBV, and LMP1 acts as a bona fide oncogene in transforming rodent fibroblast in vitro [1-4]. Structurally, LMP1 is composed of a cytoplasmic N-terminus, six transmembrane domains and a cytoplasmic C-terminus [5]. The C-terminus contains two major signaling domains, C-terminus activating regions 1 and 2 (CTAR 1, CTAR 2), which activate NF-κB [6-8] and p38 [9] signaling pathways through interaction with the effector proteins tumor necrosis factor receptor-associated factors and tumor necrosis factor receptor-associated death domain. LMP1 can also activate ERK signaling through the CTAR 1 domain [10-13], and c-jun N-terminus kinase (JNK) through the CTAR 2 domain [14,15] in an NF-κB independent manner. In addition, constitutive activation of the JAK/STAT pathway by LMP-1 has been mapped to 2 Janus kinase binding sites in the region between CTAR 1 and 2 [16,17].

We have previously shown that LMP1 increases the immunofluorescent intensity of promyelocytic leukemia nuclear bodies (PML NBs) through increased expression of PML protein [18]. The PML NB is a multifunctional proteinaceous nuclear organelle that has been implicated in transcriptional regulation, telomere maintenance, and regulation of apoptosis [19-23]. Although formation of the PML NB is dependent on the PML protein, more than one hundred different proteins have been shown to be recruited to PML NBs, which supports functional heterogeneity of the this nuclear organelle [24]. PML is induced by type I and II interferons [25,26] and has been implicated in antiviral defense [27]. Further, the conserved disruption of PML NBs by alpha, beta, and gamma herpes viruses during lytic reactivation attests to the importance of PML NBs in transcription and the herpesviral lifecycle [28-32]. Increased expression of PML contributes to the maintenance of EBV latency, while arsenic trioxide or PML siRNA disruption of PML NBs results in EBV lytic replication, affirming the role of PML in selective transcriptional control [18].

The firefly luciferase gene from *Photinus pyralis *has been sequenced, and when coupled to promoters of interest, has been a valuable tool to study transcription regulators [33,34]. The rational of the reporter assay assumes that experimental conditions modulate gene expression through binding of transcription complexes to the promoter sequence of interest (e.g.: through activation of transcription factors, etc.). However, expression of LMP1 within the cell or association with PML NBs has been shown to affect reporter activity [19,35,36]. In this study, we show that LMP1-mediated suppression of reporter activity is attributable to the upregulation of PML and PML NBs. Disruption of PML NBs by arsenic trioxide or PML siRNA restored reporter activity. These data posit a plausible explanation to reconcile previous conflicting data on LMP1 signaling activity and alerts researcher to the possibility of false-positives and false-negatives when using reporter assays as screening tools.

## Results

### LMP1 suppresses the expression of reporter plasmids

**Figure 1:**
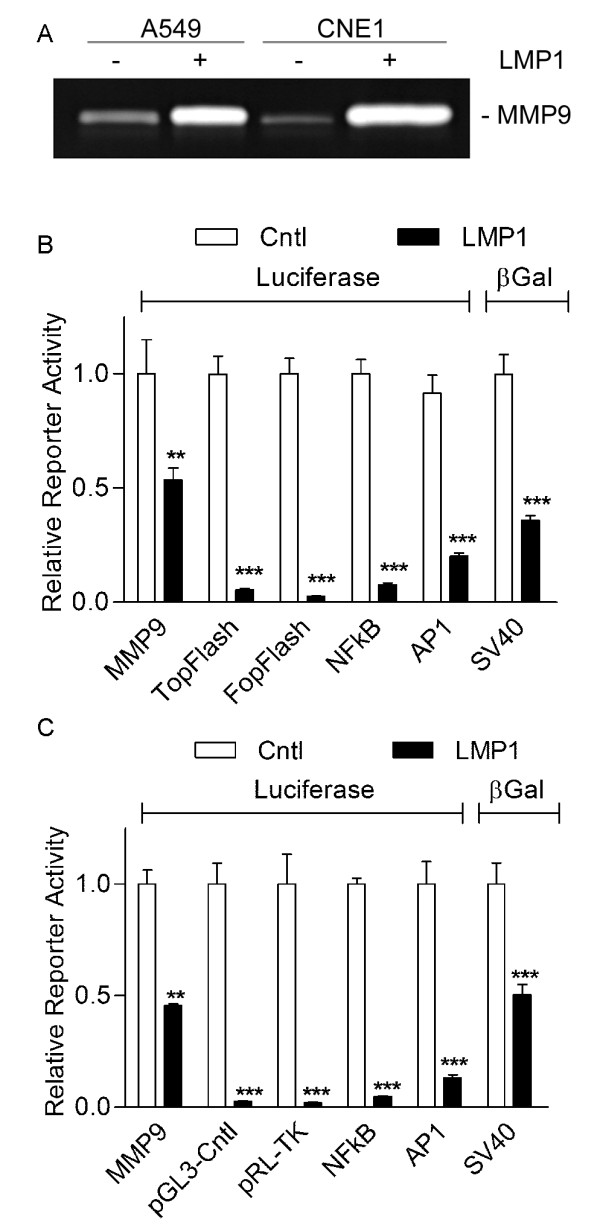
**LMP1 suppresses expression of exogenous DNA**. A) A549 and CNE1 cells retrovirally transduced with LMP1 expression or empty vector were cultured for 24 hrs. Conditioned medium was used to measure MMP9 by gelatin zymography. B) 7.5 × 10^4 ^A549 cells or C) CNE1 cells were co-transfected with 100 ng of the indicted luciferase construct and either 300 ng of empty vector or LMP1 expression vector. Cells were harvested at 24 hours post transfection.

Expression of LMP1 in A549 cells has been shown to increase transcription of the endogenous *MMP9 *gene and MMP9 protein levels in an NFκB dependent manner [37]. Consistent with previous studies, expression of LMP1 increased the levels MMP9 in condition medium (Figure 1A). In contrast, when a reporter construct containing a 700 bp fragment of the MMP 9 promoter region was co-transfected with the LMP1 expression plasmid, LMP1 expression resulted in suppression of reporter activity (Figure 1B). Selective suppression of reporter activity by LMP1 has been previously reported [35], though suppression of reporter activity in that study correlated with suppression of endogenous promoter activity. To further investigate the LMP1 induced disparity between activity on endogenous and exogenous promoters, A549 cells were co-transfected with the LMP1 expression vector and a series of reporter plasmids. Based on previous reports, LMP1 expression was expected to increase AP-1 and NF-κB responsive reporter activity while having no effect on the constitutive reporters FOP Flash, SV40, pGL3-Control and pRL-TK (renilla luciferase driven by the HSV1 thymadine kinase promoter). LMP1 expression suppressed reporter activity in assays utilizing the LEF/TCF responsive TopFlash reporter, the mutated LEF/TCF control reporter (FopFlash), an NF-κB responsive reporter, an AP1 responsive luciferase reporter construct, and a viral promoter driving β-galactosidase (SV40) (Figure 1B).

To test whether the suppression of reporter activity by LMP1 was specific to A549 cells, a similar series of reporter assays were conducted in nasopharyngeal carcinoma cells (CNE-1). LMP1 expression resulted in suppression of reporter activity in each of the constructs utilized (Figure 1C).

### LMP1 suppression of Reporter plasmids is LMP1 dose responsive

**Figure 2:**
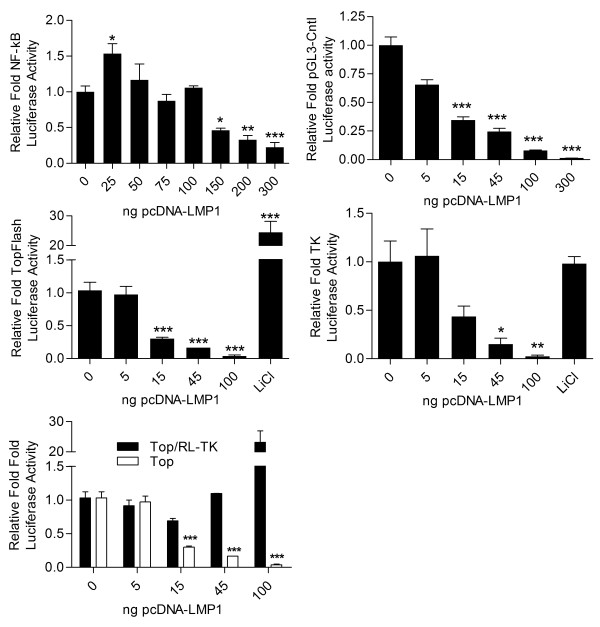
**LMP1 suppression of reporter activity is dose responsive**. 7.5 × 10^4 ^A549 cells were co-transfected with 100 ng of A) an NF-kB responsive reporter or B) the constitutive reporter pGL3-comtrol and the indicated amount of the LMP1 expression vector. The total amount of DNA in each well was held constant by addition of empty backbone vector. C & D) In a dual luciferase assay, 7.5 × 10^4 ^A549 cells were co-transfected with the TopFlash firefly and the Thymidine Kinase renilla luciferase construct and the indicated amount of the LMP1 expression vector. 10 mM LiCl was used as a positive control. Cells were harvested at 24 hours post transfection. E) Top-Flash luciferase activity normalized to RL-TK expression.

To determine that the effect was not an artifact of transfection efficiency, A549 cells were co-transfected with a constant amount of the NFκB responsive reporter along with increasing levels of the LMP1 expression vector (Figure 2A). Very low levels of LMP1 resulted in a modest increase in luciferase activity whereas increasing levels of LMP1 resulted in graded suppression of reporter activity. Reporter activity suppression was also observed when the constitutive firefly construct pGL3 (Figure 2B), the constitutive renilla luciferase reporter containing the HSV1 thymidine kinase promoter (Figure 2C), or the firefly luciferase LEF/TCF responsive TopFlash reporter (Figure 2D) were employed in a dual luciferase assay. Notably, when the LEF/TCF responsive data was normalized to the RL-TK data, an artifactual activation was observed (Figure 2E).

#### Arsenic trioxide disrupts PML NBs in LMP1 positive cells

**Figure 3:**
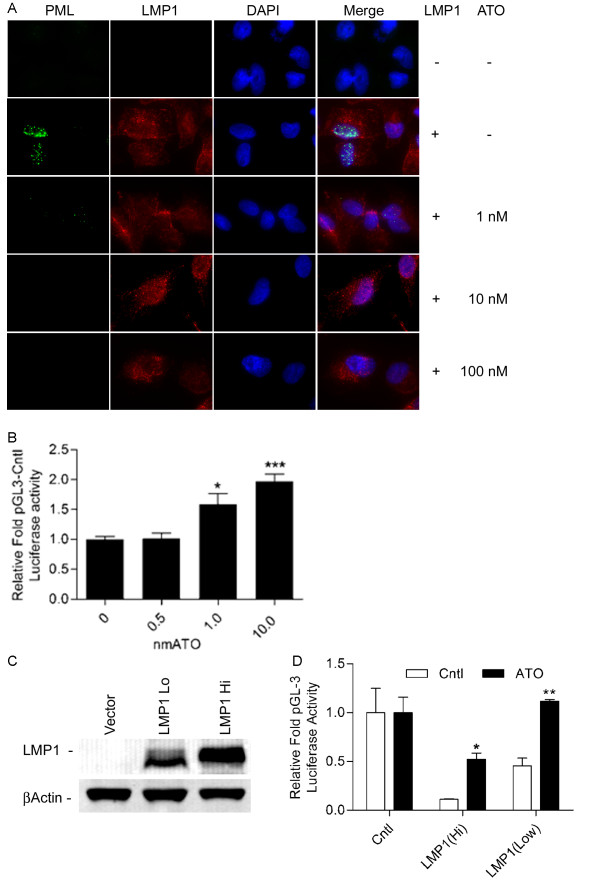
**Arsenic trioxide restores expression of exogenous DNA**. A) ATO treatment disrupts PML NB. Retrovirally transduced A549 cell expressing LMP1 or a hygromycin resistance cassette (control) were treated for 24 hours with the indicated concentration of ATO and stained for PML (green). Nuclei are stained with DAPI (blue) at original magnification of 600X. B) ATO restores luciferase activity in a dose responsive manner. 7.5 × 10^4 ^A549 < LMP1> Hi cells were transfected with 100 ng NF-kB responsive luciferase and treated with the indicated concentration of ATO for 24 hours. C) LMP1 is differentially expressed in separate retroviral transductions. A549 cells were retrovirally transduced with backbone vector expressing only the hygromycin resistance cassette or LMP1 expression vector. Western blot analysis of whole cell lysate of control cells and two separate LMP1 vector transductions; 35 ug of protein was loaded per well. Beta actin was used as a loading control. D) ATO-induced restoration of exogenous DNA expression is LMP1 dose responsive. 7.5 × 10^4 ^A549 cells retrovirally transduced with backbone vector or 2 separate retroviral transductions differentially expressing LMP1 were transfected with pGL3_Luc and treated with 8 nM ATO for 24 hours.

Arsenic trioxide (ATO) has been shown to induce polyubiquitination dependent proteasomal degradation of PML by directly binding to the conserved RBCC domain [38-40]. To determine the minimal level of ATO needed to disrupt PML NBs in A549 cells, A549 cells retrovirally transduced with LMP1 expression vector or the backbone vector were treated with 1-100 nM ATO for 24 hours and PML NBs were imaged by immunofluorescence. Treatment with ATO produced disruption of PML NBs in a dose dependent manner with complete disruption at a concentration of 10 nM (Figure 3A), whereas cytotoxcicity was noted at doses above 100 nM. To investigate whether intact PML NBs are required for LMP1 induced plasmid silencing, A549 cells transduced with an LMP1 retrovirus were transfected with the constitutive reporter pGL3 and treated with varying doses of ATO (Figure 3B). An increase in luciferase activity was seen with the addition of ATO at 1.0 and 10.0 nM concentrations, corresponding with the loss of PML NB immunofluorescence intensity.

#### ATO restored reporter activity in LMP1 positive cells

To assess the effects of LMP1 expression level on the ability of ATO to restore reporter activity, LMP1 transduced A549 cells were assessed for LMP1 expression (Figure 3C). A549 cells expressing higher and lower levels of LMP1 or transduced with the empty vector were transfected with the constitutive reporter pGL3 Control and treated with 8 nM ATO for 24. ATO treatment resulted in a 5-fold increase in reporter activity in A549 <LMP1> HI cells, though this level was below that of control. In the A549 <LMP1> Lo cells, luciferase activity was completely restored (Figure 3D).

#### Specific disruption of PML NBs by PML specific siRNA restores reporter activity

**Figure 4:**
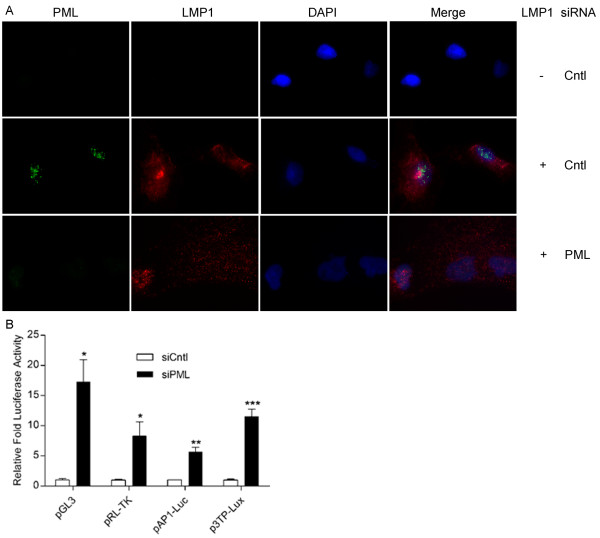
**siRNA targeted disruption of PML NBs restores MMP9 reporter activity**. A) A549 cells retrovirally transduced with either the backbone or LMP1 expression vector were transfected with either control siRNA or siRNA directed towards the PML transcript. PML NB intensity (green) was assessed at 24 hours post transfection. LMP1 is shown in red, DAPI nuclear counter-stain is shown in blue; original magnification of 400X. B) 7.5 × 104 A549 cells were reverse transfected with either the control siRNA or siRNA directed towards the *PML *transcript at plating. After 24 hours, the cells were co-transfected with 100 ng of the indicated luciferase construct and 300 ng of LMP1 expression vector. Cells were harvested at 24 hours post transfection and luciferase activity assessed.

To preclude possible off target effects by ATO in luciferase restoration assays, PML NBs were disrupted using siRNA specifically targeted to the PML transcript. Treatment of A549 cells retrovirally transduced with the LMP1 expression vector along with PML specific siRNA resulted in marked though not complete disruption of PML NBs (Figure 4A). In luciferase assays utilizing a viral (pGL3Control and pRL-TK) and cellular (pAP1-Luc and p3TP-Lux) reporters transfected into LMP1 positive A549 cells, the addition of PML specific siRNA resulted in a 7 to 17 fold increase in luciferase activity compared to control siRNA (Figure 4B).

## Discussion

A previous report demonstrated that LMP1 induces suppression of some, but not all, reporter constructs, and that reporter suppression correlated with suppression of endogenous gene expression [35]. In the present study, the suppression of reporter activity was independent of the specific promoter used. Importantly, the MMP9 reporter activity did not correlate with the observed LMP-1-mediated robust increase in endogenous MMP9 protein expression. The major activation elements in the *MMP9 *gene promoter are AP-1 and NF-κB [41], and LMP1 has been shown to increase the expression of endogenous MMP9 in an NF-κB dependent manner [37]. Co-transfection of either AP-1 or NF-κB responsive reporters along with LMP1, however, resulted in suppression of both reporters. Since LMP1 induces activation of NF-KB signaling through both CTAR1 and CTAR2 (by interacting with TRAF and TRADD) [42] it is predicted that LMP1 should increase NK-κB reporter activity in a dose responsive manner. As shown in Figure 2, however, NF-κB dependent reporter activity demonstrated a biphasic response curve resulting in suppression with moderate LMP1 input amounts and abrogation of reporter activity at higher input amounts. These findings may have significant bearing upon the EBV literature. Recently, conflicting data as to the role of LMP1 in the transcription of LEF/TCF responsive genes have been reported based on results from reporter assays [43-45]. The data contained herein may help explain the differences in reported results. When the TopFlash reporter data are normalized to RL-TK in dual luciferase reporter assays, these normalized results are inverse to the un-normalized results and show activation. Similarly, reports of LMP1 suppression of TGF-β1 responsive PAI-1 reporter constructs suggested suppression of PAI-1 gene transcription [46,47] while a previous study reported upregulation of PAI-1 by LMP1 [37,48]. The observed LMP1 suppression of reporter constructs used in this study was independent of the promoter as even the activity of minimal and constitutive promoters was suppressed. Taken together, these data suggest the possibility of false positives and false negatives when utilizing luciferase assays as a screening tool for LMP1 signaling.

The maintenance of viral latency by LMP1 has been shown to be dependent on upregulation of PML and PML NBs [18]. PML NBs have been implicated in transcriptional control [22] as well as the creation of an antiviral state [27]. Expression of LMP1 increased transcription of the endogenous *MMP9 *gene while suppressing activity of an *MMP9 *promoter driven luciferase construct, demonstrating selectivity for LMP1-mediated suppression of ectopic DNA. The suppression of ectopic DNA activity by LMP1 is dependent on upregulation of PML NB, as supported by the rescue of luciferase activity with the addition ATO or PML siRNA. The role of PML NB in transcriptional control of certain gene sets has been explored, and data suggests multiple mechanisms are involved [19,22,23]. Taken together, these data suggest PML NB's antiviral activity occurs through suppression of ectopic DNA expression.

## Materials and methods

### Cell Culture and Transfection

A549 cells were obtained from ATCC. CNE1 cells were obtained from Samuel H. Speck, (Emory University, Atlanta, Georgia, USA) and have been previously described [49]. All cells were maintained in Dulbecco's minimal essential medium (Invitrogen, Eugene Oregon) supplemented with 10% fetal bovine serum (Gemini Bio-Products, West Sacramento CA) and 10 units/ml penicillin and 10 μg/ml streptomycin (Invitrogen). For transient transfection and luciferase experiments, cells were plated in 24-well plates at an initial density of 7.5 × 10^4 ^cells per well and transfected with Lipofectamine (Invitrogen) optimized with Plus reagent (Invitrogen) according to the manufacturer's instructions. During chemical treatment, A549 and A549 derived cells were cultured in DMEM + 0.5% FBS.

### Generation of Retrovirally Transduced Cells

Generation of the pEhyg*FLAG*LMP1*wt vector and retroviral transduction of cells have previously been described [50]. Retrovirally transduced cells were maintained in 250 μg/ml hygromycin until 24 hours prior to plating for experiments. In separate infections, differential LMP1 expression was characterized and the two cell lines designated A549 <LMP1> Hi and A549 <LMP1> Lo; control cells were designated A549 <pEhyg>.

### Plasmids and Reagents

Arsenic trioxide (ATO) (Sigma Aldrich, St Louis Mo, #311383) was used at a concentration of 10 nM for treatment of A549 derived cell lines. Lithium Chloride (Sigma) was used at a concentration of 10 mM. The pcDNA*Flag*LMP1*wt vector was kindly provided by Nancy Rabb-Traub (UNC-Linberger Cancer Institute). Dominant active IκB plasmid has been previously characterized [51]and was a gift from Dean Ballard (Vanderbilt University Medical School, Nashville, TN, USA). The 700 bp promoter region MMP9 luciferase construct (MMP9-Luc) containing two AP-1 responsive sites and one NF-κB responsive site was a gift from Douglas Boyd (MD Anderson Cancer center, Houston, TX, USA), and has been previously characterized [52]. The LEF/TCF responsive reporter pSuper8TopFlash_Luc containing eight LEF/TCF responsive elements and the control, pSuper8FopFlash_Luc containing mutations in these sites were obtained from AddGene (http://www.addgene.org/) and have been previously described [53]. The NF-κB responsive reporter construct (pNFκB_Luc), control reporter plasmids pGL-3_Control luciferase and pSV40_bGal b-galactosidase were obtained from Promega (Madison, Wisconsin). The pAP1_Luc reporter construct containing multiple AP1 responsive sequences fused to a TATA-like promoter was obtained from Clonetech (Mountain View, CA).

### Luciferase Assays

For luciferase assays, 7.5 × 10^4 ^cells were plated in each well of a 24-well plate, transiently transfected as stated above with 100 ng of the specified reporter plasmid per well, and harvested at 24 hours. For co-transfection and plasmid dose response assays, the total amount of DNA was held constant between wells. Treatment with 10 mM LiCl for four hours was used as a positive control for Top-Flash luciferase activity. Luciferase activity was quantified using the Luciferase Assay System (Promega # E1500) according to manufacturer's protocol, using a single tube luminometer (EG&G Berthold Lumat LM9507) programmed for a two second delay followed by a ten second read.

### Western Blots

Nuclear and membrane fractions of indicated cell lysates were separated using the Qproteome Cell Compartment Kit (Qiagen, Valencia, CA, #37502) according to the manufacturer's directions. Briefly, 5 × 10^6 ^cells were harvested in proprietary extraction buffer, separated by centrifugation, and the cytosolic fraction (supernatant) was removed. The remaining pellet was resuspended in proprietary extraction buffer, separated by centrifugation, and the membrane fraction (supernatant) was removed. After nuclease treatment, the pellet was resuspended in 500 ul of proprietary extraction buffer, separated by centrifugation, and the nuclear fraction (supernatant) was removed. Nuclear (PML and membrane (LMP1) fractions were combined with 4X Laemmli Buffer ( 240 mM Tris, 8% SDS, 40% glycerol, 10% 2-mercaptoethanol, 0.02% bromophenol blue) and 30 micrograms of protein per well were loaded in a 10 well NuPage Mops 4-12% gradient minigel (Invitrogen). Proteins were separated by electrophoresis at 130 V for 1.5 hours. Separated proteins were transferred for 1.5 hours at 30V to PDVF membrane (Invitrogen). Protein transfer was verified by Ponsou-S staining. Membranes were blocked in 5% BSA in PBST for 1hr prior to application primary antibody overnight at 4°C while shaking at 55 rpm. Rabbit polyclonal primary antibody to PML (Santa Cruz Biotechnologies, Santa Cruz, CA, #sc-5621) was used at a dilution of 1:200. Rabbit polyclonal primary antibody to β-Actin (Cell Signaling, #4967) was used at a dilution of 1:1000. Mouse monoclonal primary antibody to LMP1 was (B D Bioscience, Bedford, MA, #559898) used at a dilution of 1:1000. The secondary antibodies were fluorophore conjugated IRDye 680 goat anti-mouse IgG (LiCor Lincoln, NE, #926-32220) or IRDye 800CW goat anti-rabbit IgG (LiCor, #926-32211), and were used at a dilution of 1:15,000. Membranes were imaged using an Odyssey Infrared Imaging System (LiCor).

### Immunofluorescence Microscopy

A549 cells were plated on 8-chambered Lab-Tek slides at 2 × 10^4 ^cells per well; chemical treatment began 24 hours after plating. At specified time points, cells were rinsed twice with PBS and fixed in 4% Paraformaldehyde (freshly diluted from 16%, Electron Microscopy Science, Hatfield, PA) for 10 min at room temperature. The cells were then rinsed twice with PBS and permeabilized with 0.5% Triton X100 (Sigma) in PBS for 15 min at room temperature. Primary antibodies were applied overnight in a humidified chamber at 4° C. Following 3 rinses in PBS, secondary antibodies (1:500 dilution) were applied for 1 hour at room temperature. The chambers were removed and Vectashield with DAPI (Vector Laboratories, Burlingame, CA # H1200) was applied with a cover slip mount. The slides were imaged using a Nikon Eclipse 80i microscope and a SensiCam QE camera (Cooke Corporation) and IPLab V3.65a software (Scanalytics). Cells were grown on the same chamber slide and were exposed to the same antibody concentration and wash times. Exposure conditions were optimized for the brightest field of the specified conditions and held constant for subsequent exposures. Primary mouse monoclonal antibody (sc-966) and rabbit polyclonal antibody (sc5621) to PML were purchased from SantaCruz and used at a 1:500 dilution. Primary antibody to LMP1 was purchased from BD Bioscience (#559898) and used at a dilution of 1:500. Secondary antibodies that were employed were Alexa Fluor 594 goat anti-mouse (Invitrogen #A11005), AlexaFluor 594 goat anti-rabbit (Invitrogen#A21207) and AlexaFluor 488 goat anti-mouse (Invitrogen #A11008) and were as used at a dilution of 1:500.

### Statistical Analysis

Individual comparisons were analyzed by two-tailed unpaired *t *tests; multiple comparisons were analyzed by ANOVA with Modified Bonferroni post hoc test. A p-value < 0.05 was considered significant. For figures, (*) denotes p < 0.05 compared to control, (**) denotes p < 0.01 compared to control, (***) denotes p < 0.001 compared to control, (#) denotes p < 0.05 ATO compared to ATO/GCV co-treatment, (###) denotes p < 0.001 ATO compared to ATO/GCV co-treatment. The presented data is representative of multiple experiments performed in triplicate. Data is represented as the mean (+/-) SEM.

## Competing interests

The authors declare that they have no competing interests.

## Authors' contributions

MS conceived of the study, and participated in its design and coordination, carried out experiments, performed data analysis and statistical analysis, GB conceived of the study, participated in its design and coordination, carried out experiments, performed data analysis. RC carried out experiments and participated in data analysis, BS participated in its study design and coordination, EF participated in its study design and coordination, JL conceived of the study, and participated in its design and coordination. All authors read and approved the final manuscript.
